# CX3CR1 is a prerequisite for the development of cardiac hypertrophy and left ventricular dysfunction in mice upon transverse aortic constriction

**DOI:** 10.1371/journal.pone.0243788

**Published:** 2021-01-07

**Authors:** Christina Katharina Weisheit, Jan Lukas Kleiner, Maria Belen Rodrigo, Sven Thomas Niepmann, Sebastian Zimmer, Georg Daniel Duerr, Mark Coburn, Christian Kurts, Stilla Frede, Lars Eichhorn

**Affiliations:** 1 Department of Anesthesiology and Intensive Care Medicine, University Hospital Bonn, Bonn, Germany; 2 Heart Center Bonn, Clinic for Internal Medicine II, University Hospital Bonn, Bonn, Germany; 3 Department of Cardiac Surgery, University Clinical Centre Bonn, Bonn, Germany; 4 Institute of Experimental Immunology, University Hospital Bonn, Bonn, Germany; Scuola Superiore Sant’Anna, ITALY

## Abstract

The CX3CL1/CX3CR1 axis mediates recruitment and extravasation of CX3CR1-expressing subsets of leukocytes and plays a pivotal role in the inflammation-driven pathology of cardiovascular disease. The cardiac immune response differs depending on the underlying causes. This suggests that for the development of successful immunomodulatory therapy in heart failure due to chronic pressure overload induced left ventricular (LV) hypertrophy, the underlying immune patterns must be examined. Here, the authors demonstrate that Fraktalkine-receptor CX3CR1 is a prerequisite for the development of cardiac hypertrophy and left ventricular dysfunction in a mouse model of transverse aortic constriction (TAC). The comparison of C57BL/6 mice with CX3CR1 deficient mice displayed reduced LV hypertrophy and preserved cardiac function in response to pressure overload in mice lacking CX3CR1. Moreover, the normal immune response following TAC induced pressure overload which is dominated by Ly6C^low^ macrophages changed to an early pro-inflammatory immune response driven by neutrophils, Ly6C^high^ macrophages and altered cytokine expression pattern in CX3CR1 deficient mice. In this early inflammatory phase of LV hypertrophy Ly6C^high^ monocytes infiltrated the heart in response to a C-C chemokine ligand 2 burst. CX3CR1 expression impacts the immune response in the development of LV hypertrophy and its absence has clear cardioprotective effects. Hence, suppression of CX3CR1 may be an important immunomodulatory therapeutic target to ameliorate pressure-overload induced heart failure.

## 1 Introduction

Left ventricular (LV) hypertrophy is a main determinant of morbidity and mortality as it occurs in response to various stimuli, including systemic arterial hypertension, aortic stenosis or remodeling of the myocardium after myocardial infarction (MI) [1]. Chronic pressure overload initiates a remodeling process which changes tissue morphology and the function of the heart [2, 3].

Currently, therapeutic approaches to influence cardiac remodeling are still limited and presume an early diagnosis and consequent therapy of the underlying pathomechanisms [4]. Many, initially protective neurohumoral and inflammatory compensatory processes which become harmful in the course of disease culminate in heart failure as the final stage of the disease [5, 6]. In this light, understanding the underlying mechanisms of the cardiac immune response and its association with cardiac tissue remodeling during chronic pressure overload conditions are necessary for the development of new therapeutic strategies.

Cardiac macrophages are known to play a crucial role in cardiac tissue remodeling and healing after injury. They are derived from yolk sac and fetal monocyte progenitors and can be distinguished based on expression of the chemokine receptor, Chemokine Receptor Type 2 (CCR2). Studies have demonstrated that CCR2^+^ cardiac resident macrophages are derived from monocytes while CCR2^−^ macrophages originate from the embryonic developmental stage [7]. In steady state, resident cardiac macrophages are replenished by local proliferation. However, following cardiac injury, cardiac macrophages are replaced by blood monocytes [8, 9]

Two major macrophage subsets differentiated in pro-inflammatory Ly6C^high^ (CCR2^high^, Cx3CR1^low^) and resident Ly6C^low^ (CCR2^low^, CX3CR1^high^) cells contribute to inflammation and tissue repair in the stressed heart. In chronic pressure overload a myeloid cell response is induced which is characterized by constant high numbers of Ly6C^low^ CX3CR1^high^ macrophages [10], in contrast to the biphasic macrophage response following myocardial infarction (MI) [11]. As to this, we were able to show, that Ly6C^low^ CX3CR1^high^ macrophages act as sentinels and display helper-cell functions, allowing neutrophil trans-epithelial migration in a murine urinary tract infection model [12], but little is known about the role of these cells in myocardial pressure overload.

The receptor CX3CR1 and its ligand fractalkine (CX3CL1) play an important role in mediating monocyte recruitment and adhesion. In case of inflammation CX3CR1 is required to mediate Ly6C^low^ monocyte patrolling and crawling and monocyte-adhesion from blood vessels [13]. In response to pressure overload, cardiac Ly6C^low^ macrophages express high surface levels of CX3CR1 and accumulate predominantly in LV tissue next to blood vessels [10]. In kidney ischemia/reperfusion injury CX3CR1 deficiency reduced the entry of monocytes into the kidney and attenuated symptoms and fibrosis [14].

Aim of this study was to examine the influence of CX3CR1 in the orchestration of the cardiac immune response in the development of LV hypertrophy.

## 2 Materials and methods

### 2.1 Mice

Female C57BL/6J mice aged 10 to 14 weeks were purchased from Charles River or bred at the central animal facilities of the medical faculty of Bonn (House of Experimental Therapy HET). *Cx3cr1*^*GFP/GFP*^ mice were originally purchased by Jackson Laboratory (Jachson Laboratory strain description B6.129P2(Cg)-Cx3cr1tm1Litt/J; stock No: 005582) and bred at the central animal facilities of the medical faculty of Bonn (HET). Mice were backcrossed at least 8 times to C57BL/6J background prior to experimental inclusion. All mice were kept under specific pathogen–free conditions in isolated, ventilated cages with free access to water and food. Mice were at rest for 1 wk in the animal facility. All animal experiments were approved by governmental ethics board at the Ministry of Nature, Environment and Consumer Protection of the German state of Northrhine Westphalia (LANUV Recklinghausen permit numbers 84–02.04.2011.A313, 84–02.04.2016.A374) and were supervised by the central animal facilities of the medical faculty of Bonn (HET, Sigmund-Freud Str. 25 in Bonn, Germany). All surgical interventions were performed under anesthesia and analgesia as described below, and all efforts were made to minimize suffering of the mice.

### 2.2 Transverse aortic constriction (TAC)

We used a closed-chest model to minimize surgical trauma performing the intervention as microinvasive as possible [15]. In short, mice where anaesthetized with isoflurane (2 Vol%), intubated with an intubation cannula (OD 1.2 mm). Respiratory rate was set to 150/min and tidal volumes to 8-10mL/kg body weight using a small animal ventilator (Harvard Apparatus; Holliston, MA). A 27 G spacer was used to standardize the degree of aortic constriction. Sham animals underwent intubation and surgery except that the suture around the aorta was not tight. For postoperative pain management buprenorphine was administered (0.1μg/g subcutaneously) every 8 h for the next three days after surgery. Mice were sacrificed 3, 6 and 21 days following surgical intervention by atlanto-occipital dislocation. The selection of the times of analysis is based on our preliminary results and mirrors the immune response following TAC composed of early and late inflammatory phase and remodelling phase [16]. We compared TAC vs. sham (control) groups and CX3CR1 deficient mice with C57BL/6J mice.

For additional illustration of the experimental setting and a schematic view of the aortic constriction model see S1 and S2 Figs.

### 2.3 Cardiac pressure–volume measurements

The cardiac function was measured under anesthesia with a 1.4 French pressure conductance catheter (SPR-839, Millar Instruments, Houston, TX) according to the manufacturer’s protocol. The catheter was inserted through the right carotid artery. After measuring the systolic blood pressure (BP), the catheter was placed into the left ventricle. Heart rate (HR), stroke volume (SV), cardiac output (CO) and ejection fraction (EF) were recorded while the amount of anesthesia was reduced to 0.8 Vol% isoflurane. These data were stored and analyzed blinded using PVAN^™^ software (Millar Instruments). The cardiac index (CI) was calculated by using CO and the body weights. The effectiveness of our TAC intervention was proven by determining the systolic pressure in the carotid artery (Table 1).

**Table 1:** CX3CR1 deficiency protects the heart from decompendation following TAC.

	A			B		
	BL6 sham n = 8	BL6 TAC [21d] n = 7		CX3CR1^*GFP/GFP*^ sham n = 6	CX3CR1^*GFP/GFP*^ TAC [21d] n = 7	
**sBP carotis, mmHg**	102 ± 20	167 ± 30	***	115 ± 24	167 ± 24	*
**HR, beats/min**	609 ± 25	594 ± 46	^ns^	603 ± 20	639 ± 16	^ns^
**EF, %**	39 ± 6	21 ± 10	**	36 ± 8	48 ± 4	^ns;^ ^#^***
**CO, μl/min**	13370 ± 1694	6054 ± 2326	****	12724 ± 2983	11348 ± 1694	^ns;^ ^#^*
**CI, μl/min/BW**	548 ± 181	212 ± 87	****	459 ± 110	461 ± 62	^ns;^ ^#^*
**ESV, μl**	35 ± 5	46 ± 11	^ns^	41 ± 7	21 ± 5	**^;^ ^#^***
**EDV, μl**	53 ± 5	51 ± 9	^ns^	57 ± 8	34 ± 6	***^;^ ^#^**
**dPdt**_**max**_, **mm Hg/sek**	15377 ± 3328	9508 ± 2654	*	11024 ± 4783	12845 ± 2670	^ns^

Part A indicating C57BL/6 data, Part B CX3CR1^*GFP/GFP*^ data; values are mean ± SD.

*p < 0.05.

**p < 0.01,

*** P<0.001; transverse aortic constriction (TAC) versus respective sham, ns = not significant versus respective sham.

^#^p indicates TAC Wt vs. TAC CX3CR1^GFP/GFP^; sBP carotis = systolic blood pressure in carotid artery; HR = heart rate; EF = ejection fraction; CO = cardiac output; CI = cardiac index; ESV = end-systolic volume; EDV = end-diastolic volume; dPdtmax is the maximal rate of rise of left ventricular pressure [21].

### 2.4 Determination of heart-weight/body-weight-index

The body weight (BW) of each mouse was measured before sacrificing the animals. The heart weight (HW) of the whole heart (without heart-auricles, pericardium, and blood) was determined after carefully explanting the organ from the murine thorax and rinsing the organ thoroughly with PBS to remove any remaining blood. The index was calculated in mg HW / g BW.

### 2.5 Flow cytometry

Immune cells were isolated from blood and LV tissue as previously described [9]. Peripheral blood (100 μl) was collected in tubes with ethylenediaminetetraacetic acid (EDTA) (Sigma Aldrich, St. Louis, MO). Erythrocytes were lysed with RBC lysis buffer (Thermo Fisher Scientific, Waltham, MA). Whole hearts were flushed with PBS, minced into small pieces using a single-edged blade, and digested in gently agitated RPMI medium with 1 mg/ml collagenase-2 (Sigma Aldrich) and 1 mg/ml DNase I (Sigma Aldrich) at 37°C for 60 min.

Unspecific Fc-receptor binding was blocked incubating the cells with antimouse CD16/32 (BD Biosciences, Franklin Lakes, NJ) for 10 min at 4°C. Cells were stained with antimouse fluorochrome-conjugated antibodies in panel-appropriate combinations in the dark for 20 min at 4°C with following antibodyclones from Thermo Fisher (Waltham, MA), BD Biosciences (Franklin Lakes, NJ) and BioLegend (San Diego, CA): CD45 (AFS98), F4/80 (BM-8), CD115 (c-fms), Ly6C (HK1.4), Ly6G (1A8), CD31 (MEC 13.3); LIVE/DEAD Fixable Dead Cell Stain Kit (Thermo Fisher). BrdU Assay was performed according to the manufacturer’s protocol using the BD APC FlowKit (BD Bioscience). BrdU was administered 18 hours before analysis. We determined absolute cell numbers by adding fixed numbers of CaliBRITE APC-beads (6μm size) (BD Biosciences) before measurement as internal reference. Additionally, LV tissue and spleen were weighted before digestion and cell numbers were normalized to 100 mg tissue respectively. Flow cytometry was performed on a BD LSR Fortessa II and data were analyzed with Flow-Jo software (BD Bioscience); (Gating strategy S3 and S4 Figs).

### 2.6 Histology

#### Sirius red staining

Hearts were fixated in 4% zinc-paraformaldehyde and embedded in paraffin, and 5 μm sections from the site of the papillary muscle insertion were stained with picrosirius red (SR), as previously described [17]. Cardiomyocyte hypertrophy was assessed using area planimetry of cross-sectioned cardiomyocytes in collagen stained sections by manual cell count of at least 200 cells/slide [3]. Images were taken with a Zeiss Axio Oberserver and evaluated with Zen-Software.

### 2.7 Real time PCR

The LV tissue samples were stored at -80°C until homogenization in Trizol according to manufacturer instructions (Thermo Fisher). After reverse transcription of 2000 ng total RNA into cDNA (High capacity cDNA Reverse Transcription Kit; Thermo Fisher) Aldolase C (Mm01298116_g1, Amplicon Length 59), BNP (Mm01255770_g1, Amplicon length 68) and 18S (Mm02601777_g1, Amplicon Length 76) cDNA expressions were quantified by qPCR using TaqMan PCR detection System (Life Technologies, Carlsbad, CA). The 18S ribosomal RNA was amplified as internal control. The amounts of specific cDNA were normalized to 18S using the Δct method and differences between treatment groups were calculated by using the ΔΔct method. Results were depicted as 2^-ΔΔct^ values with respect to the indicated controls [18, 19].

### 2.8 Cytokine and chemokine measurement

For cytokine and chemokine analysis LV tissue samples were stored at -80°C until homogenization in RIPA Buffer normalized to 100 μg total Protein utilizing a BCA Protein assay Kit (Thermo Fisher) according to the manufacturer’s protocol. Samples were employed according to the manufacturer’s protocol. Quantikine ELISA-Kits for the detection of MCP-1, IL-6, IL-1β and IL-10 (R&D Systems, Wiesbaden, Germany) were used.

### 2.9 Statistical analysis

Appropriate assumptions of data (e.g. normal distribution or similar variation between experimental groups) were examined before statistical tests were conducted. The number of experiments and the number of mice per group are provided in the figure legends. Student’s t-tests were used whenever two groups were compared, and one-way or two-way analyses of variance followed by Tukey’s test for multiple comparisons. The analysis was performed with Prism 8 (GraphPad Software, Inc. La Jolla, CA). The results are provided as mean±SD unless noted otherwise; p<0.05 was considered statistically significant.

## 3 Results

### 3.1 CX3CR1 signaling contributes to loss of cardiac function in pressure overload induced LV hypertrophy

To explore the specific role of CXCR1^high^ Ly6C^low^ macrophages in the development of LV hypertrophy, *Cx3cr1*^*GFP/GFP*^ mice were subjected to a minimal-invasive mouse model of TAC [20]. Firstly, we evaluated the impact of CX3CR1 expression for the preservation of myocardial function in our model of LV hypertrophy and assessed functional parameters by pressure-volume (PV) measurements 21 days after TAC. In our recent studies, we found that 21 days after TAC the acute phase of cardiac remodeling is completed and allows to evaluate the effect of chronic pressure overload and LV hypertrophy on myocardial performance. Hence, in the current study we focused on 21 days after TAC. One major criterium for a good quality in TAC surgery and PV catheter performance is a constant systolic blood pressure, measured in the carotid artery in TAC mice. Our data highlight this high quality of TAC-surgery and PV catheter performance, as there is no difference between Wt and *Cx3cr1*^*GFP/GFP*^ TAC animals observable at day 21 (Table 1).

**Fig 1:**
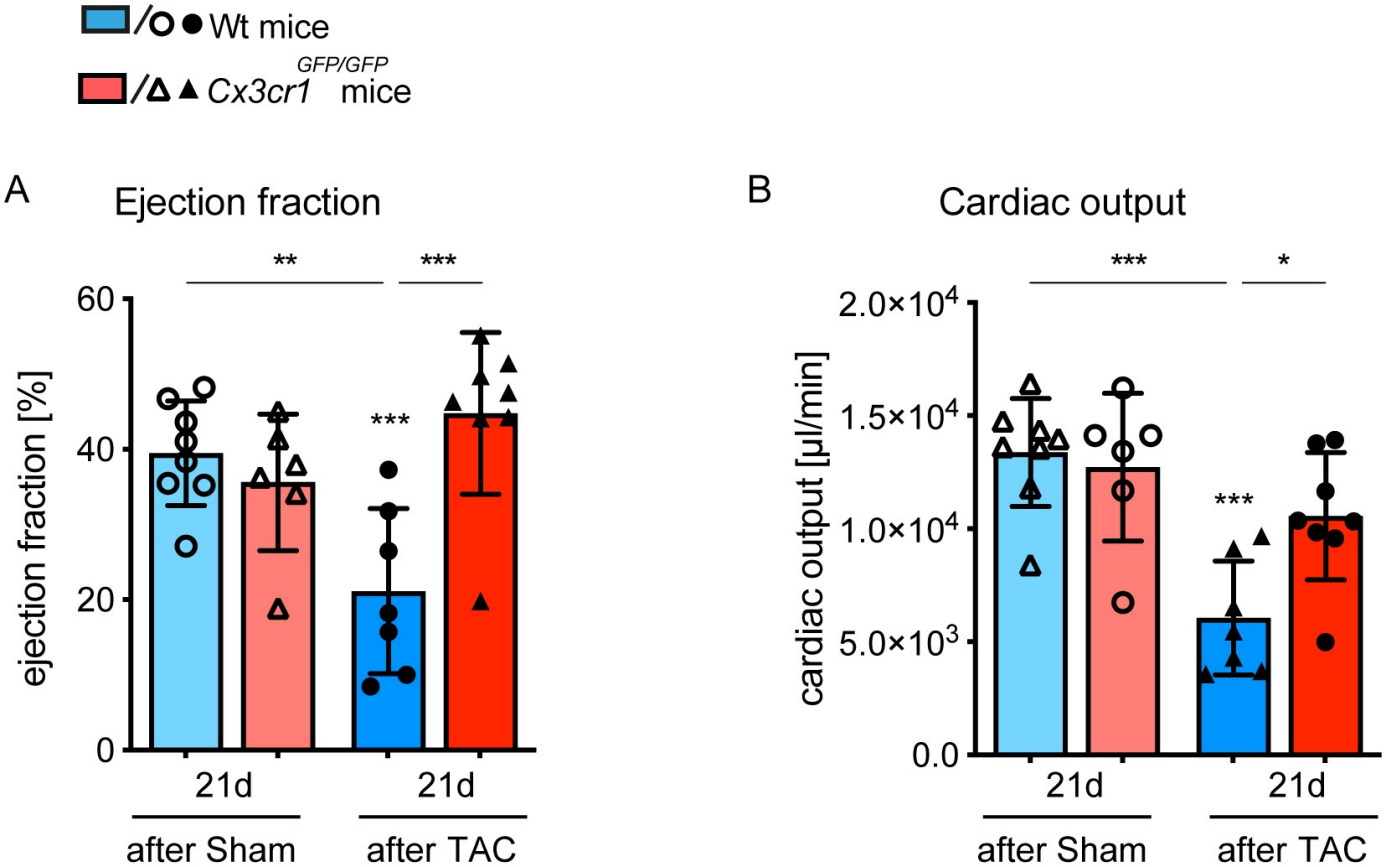
Cx3cr1^GFP/GFP^ mice exhibit a cardioprotective phenotype in response to chronic pressure overload. Using pressure volume measurement, the cardiac function of *Cx3cr1*^*GFP/GFP*^ mice and Wt mice was evaluated 21 days after TAC or sham operation. (A, B) Ejection fraction and cardiac output are depicted as plots, more information is provided in Table 1. N = 6–8 mice/group; * above individual columns indicate significant differences between TAC and respective sham group; *P <0.05, **P<0.01, *** P<0.001.

Our PV measurements reveled that CX3CR1 critically contributes to impaired cardiac function as it normally occurs in response to pressure overload. In Wt mice ejection fraction (EF) and cardiac output (CO) were significantly reduced 21 days after TAC compared to respective sham mice. In contrast, there was no reduction of EF and CO detectable in *Cx3cr1*^*GFP/GFP*^ mice (Fig 1A and 1B). Additionally, we defined the cardiac index (CO/bodyweight) relating heart performance to the size of the corresponding animal, to exclude potential influences of animal weight to the results, and observed significantly better left ventricular function in *Cx3cr1*^*GFP/GFP*^ mice compared to Wt mice 21 days after TAC (Table 1). Merely, ESV (end-systolic volume) and EDV (end-diastolic volume), parameters related to systolic and diastolic cardiac function, were significantly reduced in response to pressure overload in *Cx3cr1*^*GFP/GFP*^ mice. These findings indicate that *Cx3cr1*^*GFP/GFP*^ mice react to TAC induced pressure overload but do not develop severe impairment of LV function and signs of heart failure.

### 3.2 CX3CR1 signaling modulates the development of LV hypertrophy

**Fig 2:**
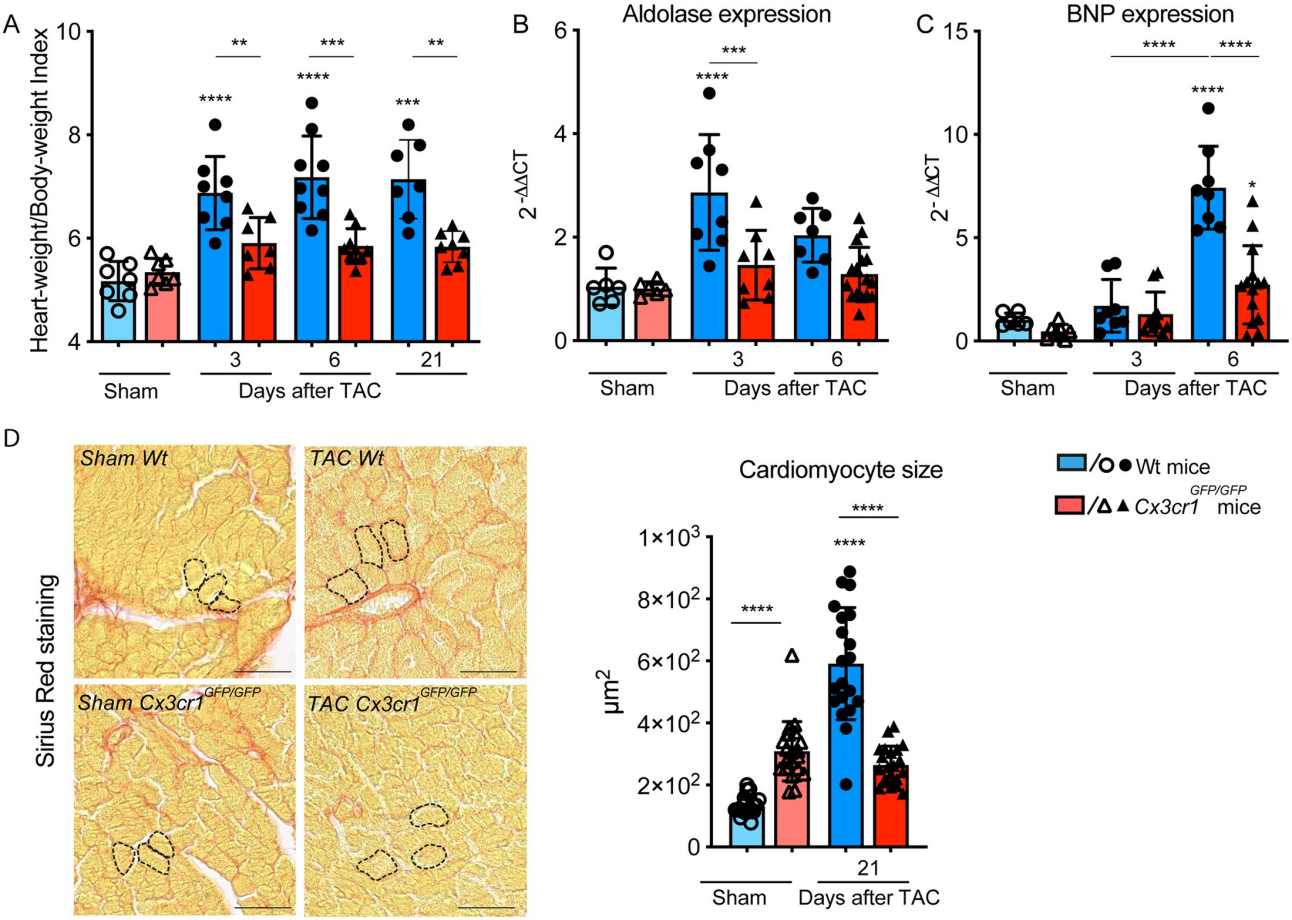
CX3CR1 expression modulates the development of LV hypertrophy in response to pressure overload. (A) Heart-Weight/Body-Weight-Index of Wt and *Cx3cr1*^*GFP/GFP*^ mice calculated 3, 6 and 21 days after transverse aortic constriction (TAC) and sham operation; n = 7–9 mice/group. (B) Aldolase mRNA expression pattern in LV tissue determined 3 and 6 days after TAC and sham operation in Wt and *Cx3cr1*^*GFP/GFP*^ mice; n = 6–14 mice/group. (C) BNP mRNA expression pattern in LV tissue determined 3 and 6 days after TAC and sham operation in Wt and *Cx3cr1*^*GFP/GFP*^ mice; n = 6–14 mice/group. (D) Representative sections of the left ventricle stained with sirious red of Wt and *Cx3cr1*^*GFP/GFP*^ mice 21 days after TAC or Sham operation to determine cardiomyocyte size. Dashed lines highlight exemplary individual cardiomyocytes; scale bar indicating 50μm. Quantification of cardiomyocyte size by area planimetry; n = 5–6 mice/group, 4 slides per mouse were randomly analyzed by manual cell count of at least 50 cells/slide and mean was used for statistical analysis. * above individual columns indicate significant differences between TAC and respective sham group; **P<0.01, *** P<0.001, ****p < 0.0001.

Heart-Weight/Body-Weight-Index (HW/BW-Index) was calculated to evaluate the development of cardiac hypertrophy. Myocardial hypertrophy represents a cardioprotective mechanism in adaptation to pressure overload, when compared to CX3CR1 competent C57BL/6 mice (Wt), *Cx3cr1*^*GFP/GFP*^ mice demonstrated with significantly less hypertrophy which did not exceed sham levels at any timepoint (Fig 2A).

These results were confirmed by determination of aldolase expression in LV tissue, whose expression activity is known to correlate with the degree of hypertrophy [22]. While Wt animals showed a significant increase of aldolase expression 3 and 6 days after TAC, the expression in *Cx3cr1*^*GFP/GFP*^ mice did not change, corroborating the finding that cardiac hypertrophy is attenuated in the absence of CX3CR1 signaling (Fig 2B). Besides, we analyzed the kinetic of brain natriuretic peptide (BNP) which is predominantly produced by cardiac atria and ventricles in response to increased cardiac stretch. Levels of BNP are powerful predictors of ventricular dysfunction and mortality [23]. Pressure overload caused a significant increase in left ventricular BNP expression at day 6 after TAC in Wt animals compared to *Cx3cr1*^*GFP/GFP*^ mice and controls respectively. *Cx3cr1*^*GFP/GFP*^ mice also presented with a significant elevation of BNP expression at day 6 after TAC compared to controls (Fig 2C). At day 3 after TAC the elevation of BNP expression did not reach the level of significance.

We shed light on the cellular level and investigated cardiomyocyte size using cross-sectional area planimetry of Picrosirius Red-stained cardiomyocytes (Fig 2D): according to the above-mentioned data, it unraveled significantly enlarged cardiomyocytes in Wt animals 21 days following TAC intervention, whereas in *Cx3cr1*^*GFP/GFP*^ mice TAC did not cause any noteworthy increase in cardiomyocyte size (Fig 2D). Taken together, these findings support our hypothesis that development of myocardial hypertrophy is modulated by CX3CR1^high^ Ly6C^low^ macrophages.

### 3.3 CX3CR1 deficiency drives an early pro-inflammatory phenotype in the cardiac immune response

**Fig 3:**
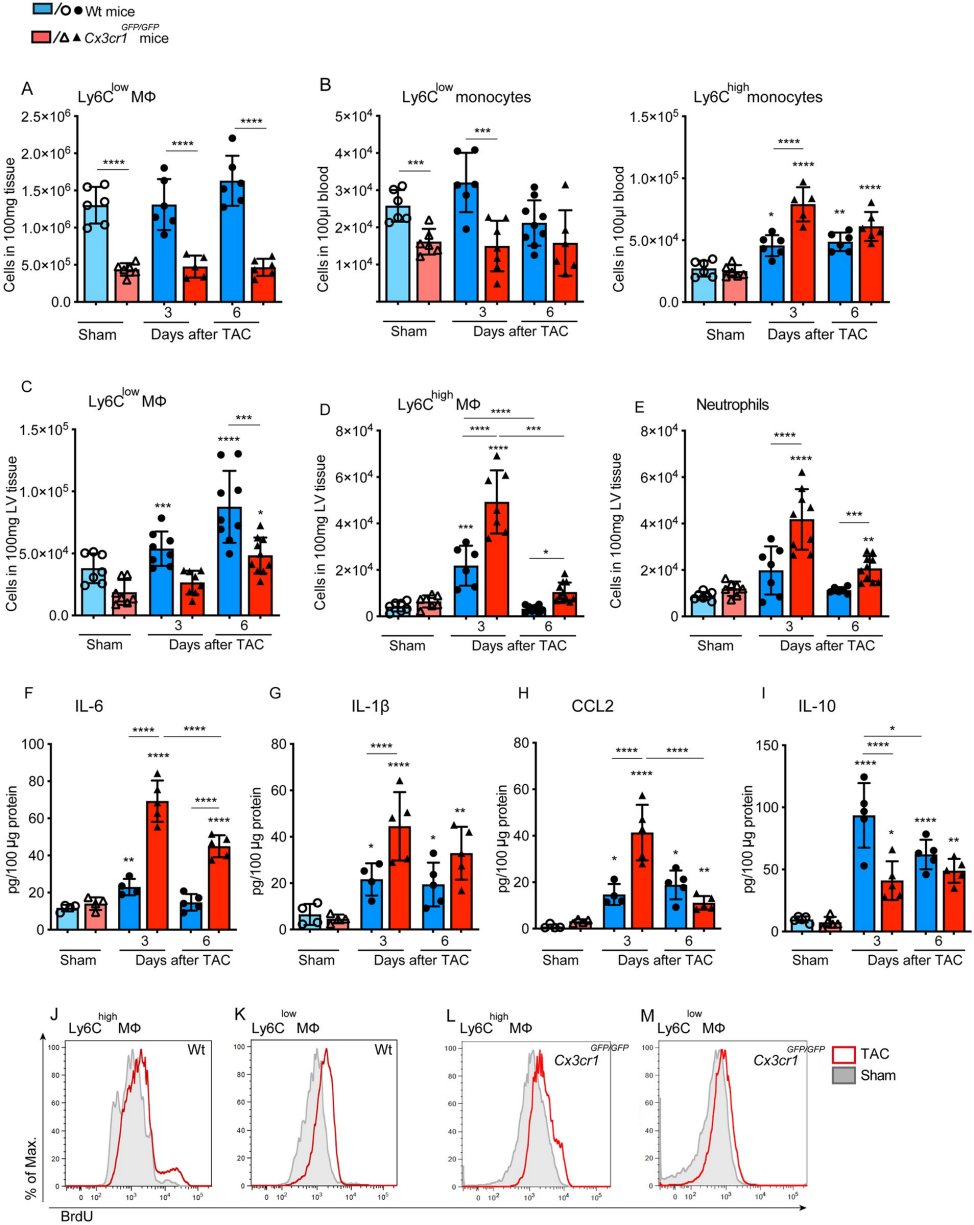
CX3CR1 deficiency drives the immune response to a pro-inflammatory phenotype during chronic pressure overload. Quantification of Ly6C^low^ F4/80^+^ macrophages in the spleen and of (B) Ly6C^low^ and Ly6C^high^ CD115^+^ monocytes in the blood of Wt and *Cx3cr1*^*GFP/GFP*^ *mice* determined by flow cytometry 3 and 6 days after TAC and sham ioperation. (C-E) Number of neutrophils, Ly6C^low^ and Ly6C^high^ macrophages in the LV tissue was determined by flow cytometry analysis in *Cx3cr1*^GFP/GFP^ and Wt mice 3 and 6 days after TAC and sham operation. The particular cell subsets were defined as follows: neutrophils as CD45^+^ F4/80^-^ Ly6G^+,^ macrophages as CD45^+^ F4/80^+^ Ly6G^-^; Ly6C^high^ and Ly6C^low^ macrophages were further discriminated according to the respective Ly6C surface expression; (F-I) Concentrations of the pro-inflammatory cytokines IL6 and IL-1β, the chemokine MCP1 and IL-10 were determined in LV tissue homogenates at day 3 and 6 after surgical intervention in *Cx3cr1*^*GFP/GFP*^ and Wt mice using ELISA assays. (J-M) BrdU content of cardiac Ly6C^high^ and Ly6C^low^ macrophages was determined by flow cytometry analysis at day 3 after TAC. BrdU was administered 18h before analysis. Histograms are based on concetenated plots of 4 mice. N = 6–8 mice/group; * above individual columns indicate significant differences between TAC and respective sham group; *P <0.05, **P<0.01, *** P<0.001, ****p < 0.0001.

Time-points for the analysis of the immune response were chosen according to our recent findings highlighting that pressure overload induced a significant accumulation of CX3CR1^high^ Ly6C^low^ macrophages within the first week following TAC intervention [10]. We determined the influence of fractalkine receptor expression in the orchestration of the cardiac immune response characterizing the reaction of the immune system in response to pressure overload in *Cx3cr1*^*GFP/GFP*^ mice starting with an overlook for monocyte dynamics in spleen and blood. In response to pressure overload, neither Wt, nor *Cx3cr1*^*GFP/GFP*^ mice displayed any significant changes in Ly6C^low^ macrophage number in the spleen compared to sham. But, comparing both genotypes, *Cx3cr1*^*GFP/GFP*^ mice present with significantly reduced Ly6C^low^ macrophage numbers indicating that the splenic reservoir of Ly6C^low^ macrophages is influenced by CX3CR1 signaling [24]. This situation is also reflected in the blood. The number of Ly6C^low^ monocytes is significantly reduced in *Cx3cr1*^*GFP/GFP*^ mice compared to Wt mice. However, CX3CR1 deficiency caused a significant increase in Ly6C^high^ monocyte numbers in the blood in response to pressure overload 3 days after TAC compared to Wt mice and respective sham (Fig 3B). Wt mice also presented with increased Ly6C^high^ monocyte numbers in response to TAC. But we observed a 3-fold increase of Ly6C^high^ monocytes in CX3CR1 deficient mice whereas in Wt mice the number of these cells increased only 1.7-fold.

In the myocardium, blocking of CX3CR1 expression disrupted the normal Ly6C^low^ macrophage driven immune response resulting in significantly increased Ly6C^low^ macrophage numbers only 6 days after TAC compared to the sham group and with considerably lower cell numbers compared to Wt (Fig 3C). Opposing to these findings, we observed increased numbers of Ly6C^high^ macrophages and neutrophils in the LV myocardium of *Cx3cr1*^*GFP/GFP*^ mice at day 3 and 6 after TAC compared to the respective Wt groups and sham control. The number of Ly6C^high^ macrophages in Wt mice was significantly elevated only at day 3 after TAC compared to sham controls and reached baseline level on day 6 whereas neutrophils did not increase significantly at any time-point (Fig 3D and 3E).

The immune reaction in the hypertrophic heart was further portrayed investigating the protein expression of cytokines and chemokines, known to mediate inflammation and leukocyte recruitment. Here, impaired CX3CR1 signaling caused an increase in pro-inflammatory cytokine levels in LV tissue. The protein concentration of IL-6 and IL1-β increased significantly in *Cx3cr1*^*GFP/GFP*^ mice compared to Wt and respective sham 3 and 6 days after TAC. In Wt mice the kinetics of IL-6 and IL1-β was less impressive, reaching the level of significance only at day 3 (IL-6) but on day 3 and 6 (IL1-β) after TAC in Wt mice compared to sham (Fig 3F and 3G).

Various molecules are involved in monocyte chemotaxis and attraction. Among these, CCL2 is modulating the recruitment of Ly6C^high^ CCR2^+^ monocytes, which is of interested with regard to Ly6C^high^ monocyte kinetics in *Cx3cr1*^*GFP/GFP*^ mice during pressure overload. Blocking of CX3CR1 signaling was accompanied by a significant increase in CCL2 levels 3 and 6 days after TAC compared to sham controls. Noteworthy, CCL2 concentration increased 4-fold on day 3 in *Cx3cr1*^*GFP/GFP*^ mice while Wt mice exhibited a weaker, but significant increase of CCL2 at the same time. While CCL2 concentration peaked on day 3 in *Cx3cr1*^*GFP/GFP*^ mice and decreased thereafter, it remained elevated in Wt mice after 6 days compared to respective sham (Fig 3H). Furthermore, the concentration of IL-10 protein in LV tissue was determined, as it is known to improve post MI repair and macrophage polarization [25]. A deficiency in CX3CR1 signaling influenced IL-10 concentration causing significantly lower IL-10 levels in the LV tissue of *Cx3cr1*^*GFP/GFP*^ mice compared to Wt mice 3 days after TAC. Though, IL-10 levels significantly increased in *Cx3cr1*^*GFP/GFP*^ mice and Wt animals at both time-points after TAC in comparison with the respective sham controls (Fig 3I).

In the next step, we investigated the origin of the increased Ly6C^high^ macrophage number in the heart tissue of *Cx3cr1*^*GFP/GFP*^ mice, which could be either driven by CCL2-CCR2 mediated recruitment from the circulation, or by local proliferation within the myocardium. Therefore, we examined local immune cell proliferation in response to pressure overload performing BrdU assays. However, we could not detect TAC induced differences in proliferation of neither Ly6C^low^, nor Ly6C^high^ macrophages in the LV tissue of *Cx3cr1*^*GFP/GFP*^ mice and Wt mice (Fig 3J–3M).

Taken together, these data highlight that CX3CR1 expression balances the immune response in the heart. A lack of CX3CR1 dependent recruited cells drives the immune response towards an early pro-inflammatory phenotype.

## 4 Discussion

Chronic pressure overload caused by systemic arterial hypertension or aortic valve stenosis initiates processes which can adversely support tissue remodeling and cardiac function. Normally, myocardial hypertrophy represents a cardioprotective mechanism in adaptation to pressure overload. However, many inflammatory mechanisms become harmful in the course of this disease potentially culminating in terminal heart failure [5, 6]. We are aware that the cardiac immune response differs depending on the underlying causes. This suggests that development of new therapeutic strategies demands a thorough understanding of the underlying mechanisms of the cardiac immune response and its correlation to cardiac tissue remodeling.

In this study, we were able to demonstrate that fractalkine receptor CX3CR1 expression is essential in the development of LV hypertrophy and heart failure in response to pressure overload.

The CX3CL1/CX3CR1 axis mediates recruitment and extravasation of CX3CR1-expressing subsets of leukocytes and plays a pivotal role in the inflammation-driven pathology of cardiovascular disease. We investigated the role of CX3CR1 in the development of pressure overload induced LV hypertrophy examining *Cx3cr1*^*GFP/GFP*^ mice, which are deficient for CX3CR1 [26]. CX3CR1 is ubiquitously expressed on most tissue macrophages and DCs but does not play a major role for their ontogeny, homeostatic migration, or colonization of tissues with resident phagocytes [27]. Our latest results showed that a pressure overload induced immune response is characterized by the accumulation of Ly6C^low^ CX3CR1^high^ macrophages labeling the immune reaction in chronic cardiac impairment [10]. The expression of CX3CR1 on other, than immune cells in the heart was excluded by flow cytometry analysis of CD45^negative^ cells (S2 Fig). We can confirm that CX3CR1 deficient mice present significantly reduced numbers of Ly6C^low^ macrophages in the spleen and Ly6C^low^ monocytes in the blood compared with Wt animals [26]. In the heart of *Cx3cr1*^*GFP/GFP*^ mice, a low number of Ly6C^low^ macrophages were accompanied by increased numbers of pro-inflammatory neutrophils and Ly6C^high^ macrophages in response to pressure overload. We suppose that the accumulation of Ly6C^high^ macrophages in the heart is due to enhanced CCL2 driven recruitment of Ly6C^high^ CCR2^+^ monocytes from the circulation in the absence of CX3CR1. This presumption is corroborated by the measurement of increased CCL2 concentrations in the homogenates of LV tissue from CX3CR1 deficient mice. A similar phenotype was observed in mice deficient for Nr4a1, an obligate transcription factor for Ly6C^low^ monocytes, in a model of myocardial infarction. Absolute numbers of Ly6C^high^ and Ly6C^low^ macrophages were increased in the myocardium on day 7, and global macrophage transcription was skewed towards a pro-inflammatory phenotype [28]. We assume that the elevated number of Ly6C^low^ macrophages in LV tissue at day 6 after TAC in CX3CR1 deficient mice points to a local conversion of recruited CCR2^+^ Ly6C^high^ monocytes to Ly6C^low^ macrophages. Further differentiation of monocyte derived macrophages in the heart can lead to a reduction in Ly6C expression and consequently to an increase in Ly6C^low^ macrophages in the tissue [28]. Our advanced flow cytometry approaches revealed that the source of Ly6C^high^ and Ly6C^low^ macrophages in the first week after TAC is not local proliferation.

The pro-inflammatory phenotype we observed in the early phase of hypertrophy development characterized by increasing pro-inflammatory immune cell numbers was supported by increased concentrations of the pro-inflammatory cytokines IL-6 and IL-1β and a reduced expression of pro-remodeling/anti-inflammatory cytokine IL-10 in myocardial tissue. The reduced levels of IL10 in the heart of CX3CR1 deficient mice might be due to the low number of Ly6C^low^ macrophages we observed. Ly6C^low^ macrophages or M2 macrophages are known to release IL-10 in response to cardiac injury to suppress inflammation and activate profibrotic processes [25]. Our data highlights that the normal immune reaction in the development of LV hypertrophy is disrupted in the absence of CX3CR1 skewing the immune response toward pro-inflammation. The evaluation of inflammation as beneficial or harmful has to be correlated to the course of disease (chronic vs. acute), the respective organ and of course the point of time the results are generated. In this project we had a kinetic which allowed us to observe the acute response, the start of remodelling and a later remodelling time point. In the context of brain ischemia, CX3CR1 deficiency exhibited beneficial effects due to the limited recruitment of Ly6C^low^ iNOS^+^ monocytes [29]. In a mouse model of myocardial infarction, the treatment with anti-Fraktalkine-antibody turned out to be cardioprotective, reducing adverse tissue remodeling and macrophage migration [30]. More research is needed to clarify the cardioprotective effect of inflammation in the development of LV hypertrophy.

Our investigations focusing on the morphology of the heart in *CX3CR1*^*GFP/GFP*^ mice elucidated that the cardiomyocytes presented with abrogated hypertrophy following TAC, which was reflected by constant HW/BW-Index, reduced levels of Aldolase and BNP and unchanged left ventricular function. These findings emphasize the hypothesis that the absence of CX3CR1 supports better adaptation of the myocardium to stress.

Taken together, our data suggests that CX3CR1 deficiency decreases susceptibility of the myocardium to hypertrophic damage, therefore preserving myocardial function; the underlying mechanisms seem to involve increased and timely inflammatory response.

CX3CR1 expression impacts the immune response in the development of LV hypertrophy and its absence has clear cardioprotective effects. Hence, suppression of CX3CR1 may be an important immunomodulatory therapeutic target to ameliorate pressure-overload induced heart failure.

## Supporting information

S1 Fig Time scale of the experimental setting. Animals were analyzed 3, 6 and 21 days following surgical intervention. PV catheter measurement was performed at day 21.
(DOCX)

S2 Fig Schematic view of transverse aortic constriction. The lumen of the aortic arch is restricted with the help of a standardized spacer and fixed with a suture.
(DOCX)

S3 Fig Exemplary gating strategy of LV tissue. Gating of cells of interest was performed according to beads granularity and size (6μm) (A) excluding trash, myocytes and myocyte fragments. Firstly, single cells were gated excluding doublets (B). Next, living cells were isolated (C). Living CD45+ immune cells (D) were differentiated according to Ly6G and F4/80 surface expression (E). Ly6G+ F4/80- neutrophils and F4/80+ macrophages were defined. Concatenated plots of 3 individual samples were used.
(DOCX)

S4 Fig Exemplary gating to define Ly6C^high^ CX3CR1^-^ and Ly6C^low^ CX3CR1^high^ macrophages in the LV tissue. Concatenated plots of 3 individual samples.
(DOCX)

## Abbreviations

BP: blood pressure
BrdU: bromodeoxyuridine
BW: body weight
CI: cardiac index
CO: cardiac outpu
EF: ejection fraction
FACS: fluorescence-activated cell sorting
HR: heart rate
IL: interleukin
LV: left ventricle
MCP1: monocyte chemoattractant protein 1
PV: pressure volume
SV: stroke volume of the left ventricle
TAC: transverse aortic constriction
Wt: wild type mice (C57BL/6)

## References

[1] Koga K, Kenessey A, Ojamaa K. Macrophage migration inhibitory factor antagonizes pressure overload-induced cardiac hypertrophy. American journal of physiology Heart and circulatory physiology. 2013;304(2):H282–93. Epub 2012/11/13. 10.1152/ajpheart.00595.2012 . 23144312

[2] Duerr GD, Heinemann JC, Kley J, Eichhorn L, Frede S, Weisheit C, et al Myocardial maladaptation to pressure overload in CB2 receptor-deficient mice. J Mol Cell Cardiol. 2019;133:86–98. Epub 2019/06/11. 10.1016/j.yjmcc.2019.06.003 . 31181227

[3] Velten M, Duerr GD, Pessies T, Schild J, Lohner R, Mersmann J, et al Priming with synthetic oligonucleotides attenuates pressure overload-induced inflammation and cardiac hypertrophy in mice. Cardiovasc Res. 2012;96(3):422–32. Epub 2012/09/15. . 22977006 10.1093/cvr/cvs280

[4] Massie BM. Novel targets for the treatment of heart failure: perspectives from a heart failure clinician and trialist. J Mol Cell Cardiol. 2011;51(4):438–40. Epub 2011/05/10. 10.1016/j.yjmcc.2011.03.016 . 21549124

[5] Kurrelmeyer K, Kalra D, Bozkurt B, Wang F, Dibbs Z, Seta Y, et al Cardiac remodeling as a consequence and cause of progressive heart failure. Clinical cardiology. 1998;21(12 Suppl 1):I14–9. Epub 1998/12/16. 10.1002/clc.4960211304 . 9853190 PMC6656235

[6] Hunt SA, Abraham WT, Chin MH, Feldman AM, Francis GS, Ganiats TG, et al 2009 focused update incorporated into the ACC/AHA 2005 Guidelines for the Diagnosis and Management of Heart Failure in Adults: a report of the American College of Cardiology Foundation/American Heart Association Task Force on Practice Guidelines: developed in collaboration with the International Society for Heart and Lung Transplantation. Circulation. 2009;119(14):e391–479. Epub 2009/03/28. 10.1161/CIRCULATIONAHA.109.192065 . 19324966

[7] O’Rourke SA, Dunne A, Monaghan MG. The Role of Macrophages in the Infarcted Myocardium: Orchestrators of ECM Remodeling. Front Cardiovasc Med. 2019;6:101 Epub 2019/08/17. 10.3389/fcvm.2019.00101 . 31417911 PMC6685361

[8] Heidt T, Courties G, Dutta P, Sager HB, Sebas M, Iwamoto Y, et al Differential contribution of monocytes to heart macrophages in steady-state and after myocardial infarction. Circ Res. 2014;115(2):284–95. Epub 2014/05/03. 10.1161/CIRCRESAHA.115.303567 . 24786973 PMC4082439

[9] Dutta P, Nahrendorf M. Monocytes in myocardial infarction. Arteriosclerosis, thrombosis, and vascular biology. 2015;35(5):1066–70. Epub 2015/03/21. 10.1161/ATVBAHA.114.304652 . 25792449 PMC4409536

[10] Weisheit C, Zhang Y, Faron A, Kopke O, Weisheit G, Steinstrasser A, et al Ly6C(low) and not Ly6C(high) macrophages accumulate first in the heart in a model of murine pressure-overload. PloS one. 2014;9(11):e112710 10.1371/journal.pone.0112710 . 25415601 PMC4240580

[11] Nahrendorf M, Swirski FK, Aikawa E, Stangenberg L, Wurdinger T, Figueiredo JL, et al The healing myocardium sequentially mobilizes two monocyte subsets with divergent and complementary functions. The Journal of Exp Med. 2007;204(12):3037–47. Epub 2007/11/21. 10.1084/jem.20070885 . 18025128 PMC2118517

[12] Schiwon M, Weisheit C, Franken L, Gutweiler S, Dixit A, Meyer-Schwesinger C, et al Crosstalk between Sentinel and Helper Macrophages Permits Neutrophil Migration into Infected Uroepithelium. Cell. 2014;156(3):456–68. Epub 2014/02/04. 10.1016/j.cell.2014.01.006 . 24485454 PMC4258064

[13] Auffray C, Fogg D, Garfa M, Elain G, Join-Lambert O, Kayal S, et al Monitoring of blood vessels and tissues by a population of monocytes with patrolling behavior. Science. 2007;317(5838):666–70. Epub 2007/08/04. 10.1126/science.1142883 . 17673663

[14] Li L, Huang L, Sung SS, Vergis AL, Rosin DL, Rose CE Jr., et al The chemokine receptors CCR2 and CX3CR1 mediate monocyte/macrophage trafficking in kidney ischemia-reperfusion injury. Kidney Int. 2008;74(12):1526–37. Epub 2008/10/10. 10.1038/ki.2008.500 . 18843253 PMC2652647

[15] Eichhorn L, Weisheit CK, Gestrich C, Peukert K, Duerr GD, Ayub MA, et al A Closed-chest Model to Induce Transverse Aortic Constriction in Mice. J Vis Exp. 2018;(134). Epub 2018/04/05. 10.3791/57397 . 29683463 PMC5933408

[16] Weisheit C, Zhang Y, Faron A, Köpke O, Weisheit G, Steinsträsser A, et al Ly6C(low) and not Ly6C(high) macrophages accumulate first in the heart in a model of murine pressure-overload. PLoS One. 2014;9(11):e112710 Epub 2014/11/21. 10.1371/journal.pone.0112710 . 25415601 PMC4240580

[17] Dewald O, Frangogiannis NG, Zoerlein M, Duerr GD, Klemm C, Knuefermann P, et al Development of murine ischemic cardiomyopathy is associated with a transient inflammatory reaction and depends on reactive oxygen species. Proc Natl Acad Sci U S A. 2003;100(5):2700–5. Epub 2003/02/15. 10.1073/pnas.0438035100 . 12586861 PMC151404

[18] Winer J, Jung CK, Shackel I, Williams PM. Development and validation of real-time quantitative reverse transcriptase-polymerase chain reaction for monitoring gene expression in cardiac myocytes in vitro. Analytical biochemistry. 1999;270(1):41–9. Epub 1999/05/18. 10.1006/abio.1999.4085 . 10328763

[19] Livak KJ, Schmittgen TD. Analysis of relative gene expression data using real-time quantitative PCR and the 2(-Delta Delta C(T)) Method. Methods. 2001;25(4):402–8. Epub 2002/02/16. 10.1006/meth.2001.1262 . 11846609

[20] Eichhorn L, Weisheit CK, Gestrich C, Peukert K, Duerr GD, Ayub MA, et al A Closed-chest Model to Induce Transverse Aortic Constriction in Mice. Journal of visualized experiments: JoVE. 2018;(134). Epub 2018/04/24. 10.3791/57397 . 29683463 PMC5933408

[21] Hamlin RL, del Rio C. dP/dt(max)—a measure of ‘baroinometry’. J Pharmacol Toxicol Methods. 2012;66(2):63–5. Epub 2012/02/14. 10.1016/j.vascn.2012.01.001 . 22326878

[22] Do E, Baudet S, Verdys M, Touzeau C, Bailly F, Lucas-Heron B, et al Energy metabolism in normal and hypertrophied right ventricle of the ferret heart. J Mol Cell Cardiol. 1997;29(7):1903–13. 10.1006/jmcc.1997.0429 . 9236144

[23] Ellmers LJ, Knowles JW, Kim HS, Smithies O, Maeda N, Cameron VA. Ventricular expression of natriuretic peptides in Npr1(-/-) mice with cardiac hypertrophy and fibrosis. American journal of physiology Heart and circulatory physiology. 2002;283(2):H707–14. Epub 2002/07/19. 10.1152/ajpheart.00677.2001 . 12124219 PMC4321891

[24] Swirski FK, Nahrendorf M, Etzrodt M, Wildgruber M, Cortez-Retamozo V, Panizzi P, et al Identification of splenic reservoir monocytes and their deployment to inflammatory sites. Science. 2009;325(5940):612–6. Epub 2009/08/01. 10.1126/science.1175202 . 19644120 PMC2803111

[25] Jung M, Ma Y, Iyer RP, DeLeon-Pennell KY, Yabluchanskiy A, Garrett MR, et al IL-10 improves cardiac remodeling after myocardial infarction by stimulating M2 macrophage polarization and fibroblast activation. Basic Res Cardiol. 2017;112(3):33 Epub 2017/04/26. 10.1007/s00395-017-0622-5 . 28439731 PMC5575998

[26] Jung S, Aliberti J, Graemmel P, Sunshine MJ, Kreutzberg GW, Sher A, et al Analysis of fractalkine receptor CX(3)CR1 function by targeted deletion and green fluorescent protein reporter gene insertion. Mol Cell Biol. 2000;20(11):4106–14. Epub 2000/05/11. 10.1128/mcb.20.11.4106-4114.2000 . 10805752 PMC85780

[27] Auffray C, Fogg DK, Narni-Mancinelli E, Senechal B, Trouillet C, Saederup N, et al CX3CR1+ CD115+ CD135+ common macrophage/DC precursors and the role of CX3CR1 in their response to inflammation. J Exp Med. 2009;206(3):595–606. Epub 2009/03/11. 10.1084/jem.20081385 . 19273628 PMC2699130

[28] Hilgendorf I, Gerhardt LM, Tan TC, Winter C, Holderried TA, Chousterman BG, et al Ly-6Chigh monocytes depend on Nr4a1 to balance both inflammatory and reparative phases in the infarcted myocardium. Circ Res. 2014;114(10):1611–22. Epub 2014/03/15. 10.1161/CIRCRESAHA.114.303204 . 24625784 PMC4017349

[29] Donnelly DJ, Longbrake EE, Shawler TM, Kigerl KA, Lai W, Tovar CA, et al Deficient CX3CR1 signaling promotes recovery after mouse spinal cord injury by limiting the recruitment and activation of Ly6Clo/iNOS+ macrophages. J Neurosci. 2011;31(27):9910–22. Epub 2011/07/08. 10.1523/JNEUROSCI.2114-11.2011 . 21734283 PMC3139517

[30] Gu X, Xu J, Yang XP, Peterson E, Harding P. Fractalkine neutralization improves cardiac function after myocardial infarction. Exp Physiol. 2015;100(7):805–17. Epub 2015/05/07. 10.1113/EP085104 . 25943588 PMC4686137

