# Supplementary material for: CX3CR1 is a prerequisite for the development of cardiac hypertrophy and left ventricular dysfunction in mice upon transverse aortic constriction
Source: PLoS One. 2021 Jan 7;16(1):e0243788. doi: 10.1371/journal.pone.0243788 (PMC7790399; doi:10.1371/journal.pone.0243788)
Supplement: S1 Fig — Animals were analyzed 3, 6 and 21 days following surgical intervention. PV catheter measurement was performed at day 21. (DOCX) [file pone.0243788.s001.docx]

**S1 Fig: Time scale of the experimental setting.**
