# Supplementary material for: CX3CR1 is a prerequisite for the development of cardiac hypertrophy and left ventricular dysfunction in mice upon transverse aortic constriction
Source: PLoS One. 2021 Jan 7;16(1):e0243788. doi: 10.1371/journal.pone.0243788 (PMC7790399; doi:10.1371/journal.pone.0243788)
Supplement: S2 Fig — The lumen of the aortic arch is restricted with the help of a standardized spacer and fixed with a suture. (DOCX) [file pone.0243788.s002.docx]

**S2 Fig: Schematic view of transverse aortic constriction.**
