# Supplementary material for: CX3CR1 is a prerequisite for the development of cardiac hypertrophy and left ventricular dysfunction in mice upon transverse aortic constriction
Source: PLoS One. 2021 Jan 7;16(1):e0243788. doi: 10.1371/journal.pone.0243788 (PMC7790399; doi:10.1371/journal.pone.0243788)
Supplement: S3 Fig — Gating of cells of interest was performed according to beads granularity and size (6μm) (A) excluding trash, myocytes and myocyte fragments. Firstly, single cells were gated excluding doublets (B). Next, living cells were isolated (C). Living CD45+ immune cells (D) were differentiated according to Ly6G and F4/80 surface expression (E). Ly6G+ F4/80- neutrophils and F4/80+ macrophages were defined. Concatenated plots of 3 individual samples were used. (DOCX) [file pone.0243788.s003.docx]

**S3 Fig: Exemplary Gating strategy of LV tissue.**
