# Supplementary material for: CX3CR1 is a prerequisite for the development of cardiac hypertrophy and left ventricular dysfunction in mice upon transverse aortic constriction
Source: PLoS One. 2021 Jan 7;16(1):e0243788. doi: 10.1371/journal.pone.0243788 (PMC7790399; doi:10.1371/journal.pone.0243788)
Supplement: S4 Fig — Concatenated plots of 3 individual samples. (DOCX) [file pone.0243788.s004.docx]

**S4 Fig: Exemplary gating to define Ly6C^high^ CX3CR1^-^ and Ly6C^low^ CX3CR1^high^ macrophages in the LV tissue.**
